# Tandem Mass Spectrometry Detection of Quorum Sensing Activity in Multidrug Resistant Clinical Isolate *Acinetobacter baumannii*

**DOI:** 10.1155/2014/891041

**Published:** 2014-07-02

**Authors:** Kok-Gan Chan, Huey Jia Cheng, Jian Woon Chen, Wai-Fong Yin, Yun Fong Ngeow

**Affiliations:** ^1^Division of Genetics and Molecular Biology, Institute of Biological Sciences, Faculty of Science, University of Malaya, 50603 Kuala Lumpur, Malaysia; ^2^Department of Microbiology, Faculty of Medicine, University of Malaya, 50603 Kuala Lumpur, Malaysia

## Abstract

Many *Proteobacteria* communicate via production followed by response of quorum sensing molecules, namely, *N*-acyl homoserine lactones (AHLs). These molecules consist of a lactone moiety with *N*-acyl side chain with various chain lengths and degrees of saturation at C-3 position. AHL-dependent QS is often associated with regulation of diverse bacterial phenotypes including the expression of virulence factors. With the use of biosensor and high resolution liquid chromatography tandem mass spectrometry, the AHL production of clinical isolate *A. baumannii* 4KT was studied. Production of short chain AHL, namely, *N*-hexanoyl-homoserine lactone (C6-HSL) and *N*-octanoyl-homoserine lactone (C8-HSL), was detected.

## 1. Introduction

*Acinetobacter* spp. are Gram-negative bacteria widely found in natural environment, such as soil, water, and vegetation. Most species, however, have also been associated with a variety of human infections. In the hospital environment, they often colonize critically ill patients, particularly those in intensive care and burn units [1, 2]. Furthermore, they cause a variety of opportunistic infections including bacteraemia, ventilator-associated pneumonia, surgical site infection, and catheter-related urinary tract infection [3]. Among them,* A. baumannii* has emerged as a highly troublesome pathogen for many institutions globally [4]. Since the 1970s, this bacterium has emerged as a formidable pathogen that is resistant to almost all currently available antibiotics via the acquisition of plasmids, transposons, and integrons bearing antibiotic resistance genes and the expression of numerous efflux pumps and porins [5]. The incidence of multidrug resistant infections has been increasing steadily, not just in hospital settings but also in the community [6]. Unfortunately, although the epidemiology of this pathogen has been extensively studied, relatively little is known about the molecular basis of its pathogenicity and virulence.

Quorum sensing (QS) is a mechanism of bacterial cell-to-cell communication that relies on the production, sensing, and response to signaling molecules called autoinducers [7–9]. Arguably, the most widely studied QS molecule in* Proteobacteria* is* N*-acyl homoserine lactone (AHL) which is synthesized by an AHL synthase (LuxI homologue). AHL binds to its cognate receptor (LuxR homologue) [10, 11]. Once bound, this AHL-LuxR complex is activated to regulate QS-mediated gene expression [12]. Most* Proteobacteria*, including bacterial pathogens, rely on AHL to regulate QS-dependent gene expression [13, 14]. QS is of special interest because it regulates,* inter alia*, virulence determinants [15, 16] in a population-dependent manner [12]. Thus, characterizing AHL production represents the key step to understanding the molecular basis of virulence factors expression [17].

In this study, we have isolated clinical isolate 4KT as an aetiological agentfrom the sputum of an elderly patient with diabetes mellitus who was admitted for stroke management but developed a chest infection during her hospital stay in the year 2012. We have then identified the clinical isolate as* A. baumannii* using matrix-assisted laser desorption/ionization-time-of-flight mass spectrometry (MALDI-TOF MS), and AHLs detection was our primary interest in order to study the QS activity in this clinical isolate. A variety of bacteria biosensors have been constructed [18, 19] to detect the presence of AHL signals by the activation of a reporter gene such as* lacZ* or* lux* or by the production or inhibition of a purple pigment in* Chromobacterium violaceum* or bioluminescence in* lux*-based* Escherichia coli* biosensors. However, the unequivocal identification of the type of AHLs produced requires analysis with high resolution analytical instruments such as mass spectrometry analysis. In the present work, high resolution liquid chromatography tandem mass spectrometry (LC-MS/MS) was used to detect the production of AHLs.

## 2. Materials and Methods

### 2.1. Bacteria, Biosensor, and Culture Conditions

*C. violaceum* CV026,* Erwinia carotovora* GS101, and* E. carotovora* PNP22 were grown aerobically in Luria Bertani (LB) broth with shaking (220 rpm/min) or on LB agar at 28°C [18, 20]. The components of LB medium were tryptone (10 g/L), yeast extract (5 g/L), and sodium chloride (10 g/L) while Bacto agar (15 g/L) was added to make LB agar. For MALDI-TOF MS analysis, clinical isolate 4KT was cultured on tryptic soy agar (TSA) (Scharlau, Spain) at 37°C. The composition of the TSA includes casein peptone (15 g/L), soy peptone (5 g/L), sodium chloride (5 g/L), and agar (15 g/L).

### 2.2. Isolation and Identification of Clinical Isolate

Clinical isolate 4KT grew as non-lactose-fermenting colonies on MacConkey agar and was identified as* Acinetobacter *sp. by standard microbiological tests. This identification was subsequently confirmed as* A. baumannii *by MALDI-TOF MS analysis as described previously [21]. Briefly, by using formic acid-ethanol extraction method, clinical isolate 4KT was placed onto the MSP 96 target polished steel BC plate and subjected to Microflex MALDI-TOF (Bruker Daltonik GmbH, Leipzig, Germany) bench-top mass spectrometer (equipped with UV laser at wavelength of 337 nm) with the Bruker FlexControl software version 3.3 (Build 108). Routine antibiotic susceptibility testing with the agar disk diffusion (Kirby-Baur) test showed* in vitro* resistance to aminoglycosides, fluoroquinolones, first to fourth generations of cephalosporins, beta-lactam beta-lactamase inhibitor combinations, imipenem, cotrimoxazole, and tetracyclines. The only antibiotic showing some* in vitro* activity was polymyxin B.

### 2.3. Detection of* N*-Acyl Homoserine Lactones (AHLs) Synthesis in Clinical Isolate 4KT

The AHLs biosynthesis activity of clinical isolate 4KT was assayed by perpendicular streaking with biosensor CV026 on LB agar [18].* E*.* carotovora* GS101 and* E*.* carotovora* PNP22 were used as positive control and negative control, respectively, in this study [20].

### 2.4. AHL Extraction

Clinical isolate 4KT was grown overnight in 15 mL LB medium buffered with 50 mM 3-[*N*-morpholino] propanesulfonic acid (MOPS) at pH 6.5 to prevent spontaneous lactonolysis of AHLs [22]. The supernatant was extracted twice with equal volumes of acidified ethyl acetate (0.1% v/v glacial acetic acid). Extract was concentrated to complete dryness under vacuum and resuspended with 1 mL of acetonitrile (ACN) for LC-MS/MS analysis.

### 2.5. Synthetic AHLs

**Figure 1:**
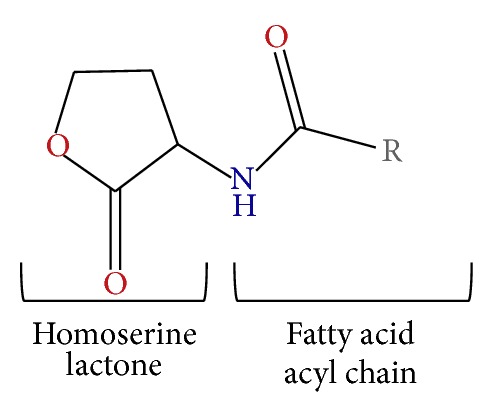
General structure of* N*-acyl homoserine lactone (AHL), where R represents the various acyl side chains.

All synthetic AHLs used in this procedure were purchased from commercial sources, Sigma Aldrich and Cayman (Michigan, USA). The structure of AHLs was shown in Figure 1. These AHLs included* N*-butanoyl-homoserine lactone (C4-HSL),* N*-hexanoyl-homoserine lactone (C6-HSL),* N*-octanoyl-homoserine lactone (C8-HSL),* N*-decanoyl-homoserine lactone (C10-HSL),* N*-dodecanoyl-homoserine lactone (C12-HSL),* N*-tetradecanoyl-homoserine lactone (C14-HSLs),* N*-(3-oxohexanoyl)-homoserine lactone (3-oxo-C6-HSL),* N*-(3-oxooctanoyl)-homoserine lactone (3-oxo-C8-HSL),* N*-(3-oxodecanoyl)-homoserine lactone (3-oxo-C10-HSL),* N*-(3-oxododecanoyl)-homoserine lactone (3-oxo-C12-HSL), and* N*-(3-oxotetradecanoyl)-homoserine lactone (3-oxo-C14-HSL). AHL stock solutions used as standards were prepared by dissolving in ACN (Merck, Frankfurt, Germany) at a concentration of 1 g/L. The stock solutions were kept at −20°C as standards in the LC-MS/MS analyses.

### 2.6. AHL Detection by LC-MS/MS

Separation of AHLs extract was performed with Agilent 1290 Infinity LC system (Agilent Technologies Inc., USA) with the HPLC column SB (C18 column, 2.1 mm × 50 mm, 1.8 *μ*m particle size, Agilent Technologies Inc., USA). Sample elution was carried out for 15 min at a constant flow rate of 0.5 mL/min at 37°C column temperature. The injection volume was at 2 *μ*L. Mobile phase A was 0.1% v/v formic acid in ultrapure water and mobile phase B was 0.1% v/v formic acid in ACN. The gradient profiles for the UPLC condition were fixed at (time: mobile phase A : mobile phase B) 0 min: 80 : 20, 7 min: 50 : 50, 12 min: 20 : 80, and 14 min: 80 : 20.

**Figure 2:**
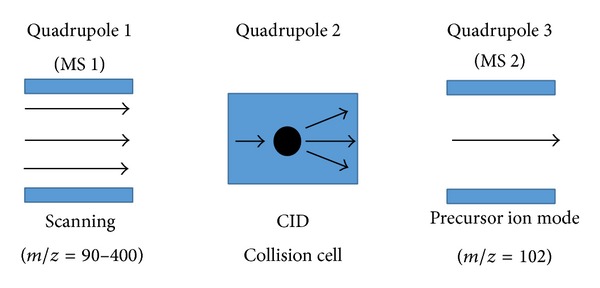
Precursor ion experiment using Agilent 6490 Triple Quadrupole LC/MS system.

MS detection of the separated compounds from UPLC was conducted using the Agilent 6490 Triple Quadrupole LC/MS system (Agilent Technologies Inc., USA). The ion source involved electrospray ion (ESI) used in positive mode. Precursor ion experiment was performed (Figure 2) for the detection of AHLs in which the product ion* m/z *was set as 102 indicating the [M+H]^+^ ion of the core lactone ring moiety [23]. The* m/z* value of the precursor ions was then scanned from 90 to 400, thus allowing the identification of various AHLs based on the detection of the core HSL moiety fragmented in the collision cell. The Agilent MassHunter software was subsequently used for the MS data analysis. Analysis was performed by comparison of retention index and extracted ion (EI) mass spectra with synthetic AHL compounds.

## 3. Results and Discussion

**Figure 3:**
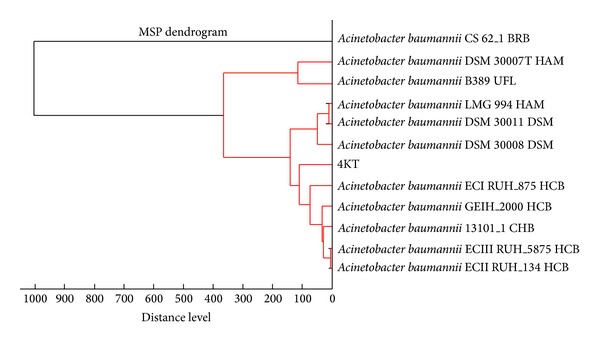
Score-oriented dendrogram of* A. baumannii *clinical isolate 4KT (labeled as 4KT in the dendrogram).

*A. baumannii* is one of the most common opportunist pathogens in healthcare settings globally [4]. It appears to have a propensity for developing antimicrobial resistance and causing serious therapeutic problems. Diabetic patients and other immunocompromised individuals are at higher risk of developing infections by this organism than the rest of the population [24]. Clinical isolate 4KT from a diabetic patient with a hospital-acquired infection was identified as* A. baumannii *with a MALDI Biotyper score of 2.384 in the best match and 2.337 in the second best match. The score-oriented dendrogram of 4KT (Figure 3) clearly shows that this isolate belongs to the species* A. baumannii*.

**Figure 4:**
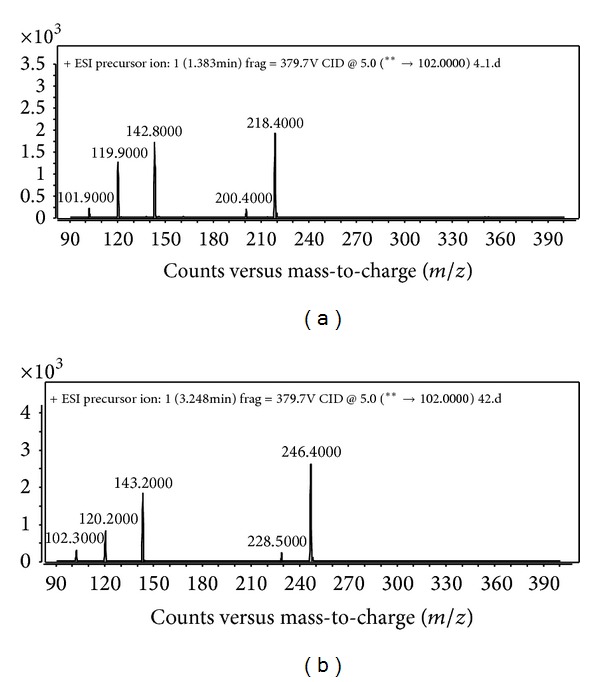
Mass spectrometry analysis of AHL produced by* A. baumannii* clinical isolate 4KT. (a) C6-HSL: retention time: 1.144 min;* m/z *200.400; abundance: 377.04; abundance%: 17.98. (b) C8-HSL: retention time: 3.105 min;* m/z* 228.100; abundance: 236.74; abundance%: 13.44.

QS activity in* A. baumannii* has been described previously [25]. Niu et al. (2008) [26] reported AHL synthase (*AbaI*) directed production of* N*-(3-hydroxydodecanoyl)-HSL (3-hydroxy-C12-HSL) along with other minor AHLs in an* A. baumannii *opportunistic pathogen. Besides that, Chan and coworkers [27] showed the production of 3-oxo-C12-HSL and 3-hydroxy-C12-HSL in an environmental* Acinetobacter* sp. strain GG2. In this study, preliminary QS screening with biosensor CV026 indicated 4KT did not produce short chain AHLs since no purple pigmentation was observed (data not shown). However, using LC-MS/MS, C6-HSL (*m/z 200.400*), and C8-HSL (*m/z* 228.500) was detected from AHLs extract from 4KT (Figure 4). Though many AHLs profiles of* A. baumannii* have been studied previously; to the best of our knowledge, this is the first report of AHL profiles in a multidrug resistant* A. baumannii*.

Discrepancy in AHL production profile in different bacterial strains belonging to the same species is not peculiar to* A. baumannii.* It has been reported that nosocomial* Acinetobacter *clinical isolates showed a distinct induction pattern that is different from the AHL profile of environmental isolates of* A*.* calcoaceticus* [28]. This difference of AHL production profile could also be due to the composition of the bacterial growth medium used and the duration of incubation [25]. It has been reported that bacteria cultured in minimal medium displayed one or two more signals, as compared to complex medium, and most bacteria showed AHL production in the stationary phase, perhaps due to the highest population cell density at this stage [25]. Besides, Middleton and coworkers [29] had proven that the AHL production profile showed variation for AHLs isolated directly from the sputum samples from patients comparing with AHLs extracted from* in vitro* analysis. In our study, the AHLs were extracted* in vitro* from isolated* A. baumannii*.

Recently, many researchers have reported on the potential use of QS as a target for the development of anti-infective therapy [30–37]. The discovery of AHLs production in multidrug resistant bacteria is important as it opens another therapy option in addition to normal antibiotic treatment which is ineffective in multidrug resistant bacteria. Quorum quenching, or interference of QS, has gained much attention due to its nonantibiotic application to attenuate bacterial infections [38–40]. The identification of AHLs in pathogens such as* A. baumannii *clinical isolate 4KT will allow screening for biomimetic or chemical analogs that can block QS mechanisms. As these molecules are mostly not antibiotics, they will not induce antibiotic resistance. This approach has been taken by Stacy and coworkers [34] who used nonnative ligands containing an aromatic aryl group closely similar to the 3-hydroxy-C12-HSL that blocks the AHL QS system in* A. baumannii*. The AHLs (C6-HSL and C8-HSL) produced by* A. baumannii *clinical isolate 4KT represent another group of AHLs that may be used for the discovery of nonnative ligands that can act as novel therapeutic drugs. This will hopefully lead to a wider spectrum of anti-QS drugs for the treatment of infections by multidrug resistant* A. baumannii*. Quorum quenching, however, only interrupts the QS activity among the bacteria and does not kill the bacteria, hence minimizing the multidrug resistance pressure; thus the approach should be applied together with the use of other drugs for synergistic effects to combat multidrug resistant pathogens.

## 4. Conclusions

Our work has shown the detection of QS activity in a multidrug resistant* A. baumannii *clinical isolate 4KT by using high resolution mass spectrometry. The production of QS signaling molecules, C6-HSL and C8-HSL, in this clinical isolate was found. This work will facilitate the search for QS-mediated genes in this pathogen and possibly pave the way for the development of anti-QS drugs to combat multidrug resistant* A. baumannii*.
